# Comment on methodological shortcomings of an analysis evaluating the eat-lancet healthy reference diet and type 2 diabetes incidence by Lopez et al.

**DOI:** 10.1038/s41430-024-01418-8

**Published:** 2024-02-23

**Authors:** Allison L. Unger, Christopher J. Cifelli, Katie Brown

**Affiliations:** National Dairy Council, Rosemont, IL USA

**Keywords:** Diseases, Scientific community

## To the Editor

We have read with interest the recent article by Lopez et al. [1], published in the *European Journal of Clinical Nutrition*. While we value the authors’ efforts to investigate the association of the EAT-Lancet commission’s recommended “Healthy Reference Diet” (EAT-HRD) and its individual dietary components with type 2 diabetes (T2D) incidence within the Mexican Teachers’ Cohort, we wish to remark on the following concerns related to the insufficient analysis and interpretation of the study’s results.

Our primary concern lies in the authors’ choice to analyze participant adherence to each dietary component of the EAT-HRD score as a binary variable (i.e., adherence versus non-adherence), rather than as a continuous variable. The authors allow by their own admission that this type of simplistic analysis may be a potential confounder, as it disregards participants who are nearly adherent to the recommended diet and categorizes them as virtually non-adherent. Yet, the implications of utilizing this analytical framework on the results presented were not contextualized or discussed in enough detail. For example, in this study, adherence to the EAT-HRD recommended target cut-off for dairy consumption in this cohort (i.e., consuming no more than 250 g of whole milk (approximately 1 cup) or 153 kcal of dairy equivalents such as yogurt or cheese per a reference diet of 2500 kcal/day [2]) was associated with increased T2D incidence. However, the amount of dairy consumed by this cohort was observed to be quite variable, with dairy consumption well above the target cut-off amount of 250 g per day in a proportion of the participants. Thus, these results suggest that consuming a greater amount of dairy each day could in fact be associated with a reduced risk of T2D incidence. Additional testing to evaluate consumption of the individual EAT-HRD dietary components, e.g. as continuous variables, would have more fully elucidated their relationship with T2D incidence in this cohort. A similar relationship was observed between added sugars and T2D incidence, yet again the authors failed to adequately discuss or explain these observations within the context of the limitations of their chosen analysis in this study.

Importantly, this lack of complete analysis leaves much interpretation to the reader on whether consumption of particular foods as part of a dietary pattern that follows the EAT-HRD is positively or negatively associated with T2D incidence. In the U.S., the 2020-25 Dietary Guidelines for Americans recommend adults consume three servings of low-fat or fat-free dairy (milk, yogurt or cheese) per day, based on the large body of evidence that these dairy foods as part of a healthy eating pattern reduce risk of chronic diseases like heart disease, T2D, obesity and certain types of cancer [3]. Out of the 94 countries globally that have food-based dietary guidelines, ~70% recommend the consumption of dairy as its own food group for health promotion [4]. Similarly, authoritative bodies align in evidence-based recommendations for a dietary pattern that limits added sugars to support health and wellness [3, 5]. In contrast, few studies have assessed the impact of the dietary pattern proposed by the EAT-Lancet commission [2] on health outcomes, while a recent analysis conducted by Beal et al. [6] revealed that the EAT-HRD recommendations would lead to deficiencies in several key micronutrients, particularly for women of reproductive age. Thus, while we applaud the authors for their efforts to investigate this understudied dietary pattern, a more robust analysis and contextualization of the reported data, particularly in a research area with a limited and mixed body of evidence, would provide a fuller understanding of the study’s conclusions and would minimize speculation by the reader.

There is a critical need for the transformation of the global agrifood environment to realize sustainable food systems on a planetary scale [7]. To support the evolution of food production with consideration of human health and finite natural resources, a robust body of evidence that provides an accurate and comprehensive understanding of how dietary choices contribute to a sustainable global food system is requisite. As researchers invest in addressing these important questions, careful and thorough investigation and reporting of the data is essential to reduce stakeholder debate and misperceptions and provide science-based solutions to achieve future public and planetary health.

## References

[1] López GE, Batis C, González C, Chávez M, Cortés-Valencia A, López-Ridaura R (2023). EAt-lancet healthy reference diet score and diabetes incidence in a cohort of Mexican women. Eur J Clin Nutr.

[2] EAT-Lancet Commission. Food Planet Health. Healthy diets from sustainable food systems summary report of the EAT-Lancet Commission. Lancet. Available from: 2019 https://eatforum.org/content/uploads/2019/07/EAT-Lancet_Commission_Summary_Report.pdf. 10.1016/S0140-6736(18)31788-4 30660336

[3] U.S. Department of Agriculture and U.S. Department of Health and Human Services. Dietary guidelines for Americans, 2020-2025. 9th Edition; https://www.dietaryguidelines.gov/.

[4] Comerford KB, Miller GD, Boileau AC, Masiello Schuette SN, Giddens JC, Brown KA (2021). Global review of dairy recommendations in food-based dietary guidelines. Front Nutr.

[5] Guideline: Sugars intake for adults and children. Geneva: World Health Organization; chrome-extension://efaidnbmnnnibpcajpcglclefindmkaj/https://iris.who.int/bitstream/handle/10665/149782/9789241549028_eng.pdf?sequence=1. (2015). 25905159

[6] Beal T, Ortenzi F, Fanzo J (2023). Estimated micronutrient shortfalls of the EAT–Lancet planetary health diet. Lancet Planet Health.

[7] Beal T (2021). Achieving dietary micronutrient adequacy in a finite world. One Earth.

